# Multiplex assay for the simultaneous detection of antibodies against small ruminant lentivirus, *Mycobacterium avium* subsp. paratuberculosis, and *Brucella melitensis* in goats

**DOI:** 10.14202/vetworld.2023.704-710

**Published:** 2023-04-08

**Authors:** Héctor D. Nájera-Rivera, Ana D. Rodríguez-Cortez, María G. Anaya-Santillán, Efrén Díaz-Aparicio, Ariadna V. Ramos-Rodríguez, Irlanda J. Siliceo-Cantero, Norma C. Vázquez-Franco, Erik Nieto-Patlán, Alejandro De Las Peñas, Liliana M. Valdés-Vázquez, Laura Cobos-Marín

**Affiliations:** 1 Department of Reproduction, Veterinary School, UNAM, Mexico City, Mexico; 2 Department of Microbiology and Immunology, Veterinary School, UNAM, Mexico City, Mexico; 3 CENID, INIFAP, Mexico City, Mexico; 4 Division of Molecular Biology, IPICYT, San Luis Potosi, Mexico

**Keywords:** antibody detection, brucellosis, Luminex^®^, paratuberculosis, serological test, small ruminant lentivirus

## Abstract

**Background and Aim:**

Brucellosis, paratuberculosis (PTb), and infections caused by small ruminant lentivirus (SRLV), formerly known as caprine arthritis encephalitis virus (CAEV), adversely affect goat production systems. Nonetheless, commonly used diagnostic tests can only determine one analyte at a time, increasing disease surveillance costs, and limiting their routine use. This study aimed to design and validate a multiplex assay for antibody detection against these three diseases simultaneously.

**Materials and Methods:**

Two recombinant proteins from the SRLV (p16 and gp38), the native hapten of *Brucella melitensis*, and the paratuberculosis-protoplasmic antigen 3 from *Mycobacterium avium* subsp. paratuberculosis (MAP) were used to devise and assess a multiplex assay. Conditions for the Luminex^®^ multiplex test were established and validated by sensitivity, specificity, repeatability, and reproducibility parameters. Cut-off points for each antigen were also established.

**Results:**

The 3-plex assay had high sensitivity (84%) and specificity (95%). The maximum coefficients of variation were 23.8% and 20.5% for negative and positive control samples, respectively. The p16 and gp38 SRLV antigens are 97% and 95%, similar to the CAEV sequence found in GenBank, respectively.

**Conclusion:**

The multiplex test can be effectively used for the simultaneous detection of antibodies against SRLV, MAP and *B. melitensis* in goats.

## Introduction

Small ruminant lentivirus (SRLV) infections, brucellosis, and caprine paratuberculosis (PTb) cause substantial farming and economic losses in goat production systems [1–3]. SRLV causes a persistent lentiviral infection in goats that has multiple clinical presentations affecting both kids and adults within a herd. There is no vaccine or specific treatment for SRLV infection [4, 5]. Brucellosis is found globally in livestock and is considered as one of seven unattended zoonoses in developing countries by the World Health Organization [6, 7, 8]. PTb is also a worldwide endemic disease that infects livestock regularly. Its causative agent, Mycobacterium avium subsp. paratuberculosis (MAP), is shed in feces, milk, and colostrum and may be spread from animal to human hosts by water and foodborne transmission routes, correspondingly representing a significant risk to public health safety [9]. Effective serological diagnostic tools are essential for accurate diagnosis of these diseases and the starting point for efficient epidemiological surveillance, as well as for the establishment of control and eradication programs.

At present, available serological tests for SRLV, brucellosis, and PTb can only identify antibodies against one pathogen at a time. Routine use of multiple single detection tests increases overall production costs and labor. Furthermore, large sample volumes are needed for each measurement (50–200 μL) [10, 11], which reduces their regular use by farmers [5, 7, 9]. Furthermore, commercial tests are mainly based on immunosorbent techniques (enzyme-linked immunosorbent assay [ELISA] and its chemiluminescence variants) that can be costly and use toxic reagents that require careful handling and proper disposal [12].

Detection methods that can simultaneously measure antibodies for multiple infectious agents have been previously developed, effectively attaining an increase in serological test efficiency [10, 13, 14]. Relevant systems include: (a) The Meso Scale Discovey method, which nonetheless has limited reproducibility and uses a toxic (carcinogenic) component in its electrochemiluminescent detection system [13]; (b) the FAST Quant technique, which includes chemiluminescent reagents that provide high sensitivity, but also has high variability, reducing its reliability [10]; and (c) the xMAP Luminex^®^ system (Luminex^®^ Corporation, Texas, USA), with a sensitivity that is superior to that observed with the ELISA technique [15]. Moreover, the Luminex^®^ multiplex system is highly reproducible and needs a small amount of sample to identify several analytes, thus lowering costs and processing time. In addition, the Luminex^®^ multiplex system does not generate toxic residues [10, 11, 13–16].

This study aimed to design and validate a Luminex^®^ multiplex assay for the simultaneous detection of antibodies against SRLV, MAP, and *Brucella melitensis* in goats, to provide an efficient diagnostic tool as the onset for the establishment of control and eradication programs.

## Materials and Methods

### Ethical approval

No human or animal subjects were used in this study so, no ethical approval was needed.

### Study period and location

The study was conducted from April 2022 to August 2022 at Veterinary School of National Autonomous University of Mexico (UNAM) with samples from a goat sera bank collected from 2017 to 2020.

### Antigens

**Figure-1:**
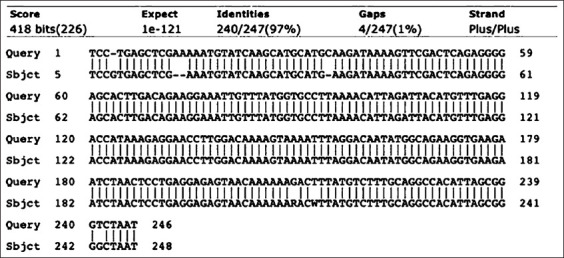
The nucleotide sequence of p16 is 97% identical to the caprine arthritis encephalitis virus genome.

**Figure-2:**
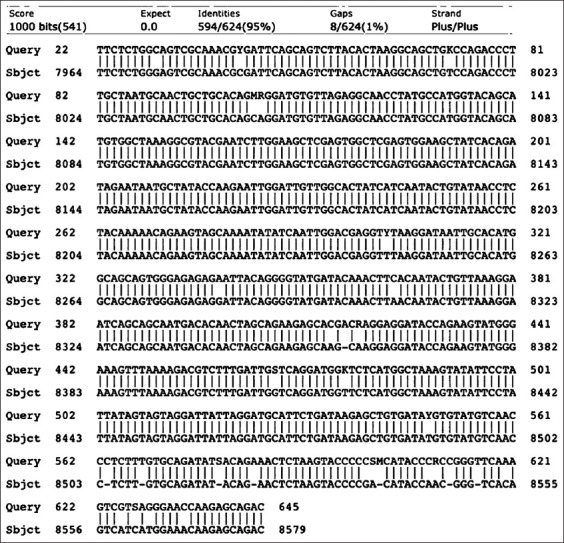
The nucleotide sequence of gp38 is 95% identical to the caprine arthritis encephalitis virus genome.

Genes encoding the p16 and gp38 proteins from SRLV were amplified by PCR using specific primers (p16 Forward 5’ GGA TCC GAA GGA GAT ATA CAT ATG GTG AGT CTA GAT AGA GAC 3’/Reverse 5’ CTC GAG TCT CCC TCC TGC TGC TTG CAC 3’) (gp38 Forward 5’ GGA TCC GAA GGA GAT ATA CAT ATG GGC GTT GGC TTG GTC ATT ATG 3’/Reverse 5’ GAG CTC TTG TCC TCT TTA GCC CAT GTC TC 3’) and the pCAEVneo11 plasmid containing the SRLV pro-viral sequence as a template [17, 18]. The PCR products were cloned in a pMB11 vector, subsequently sequenced [19] and compared with the p16 and gp38 data from GenBank (Sequence ID: M33677.1) (Figures-1 and 2). Copies were then subcloned in a pET24 expression vector (Novagen, Istanbul, Turkey) and an *Escherichia coli* BL21–Codon Plus (DE3)–RIL^©^ (*E. coli* B F^–^ *ompT* *hsdS*(rB- mB-) *dcm*^+^ Tet^R^ *gal* *endA* *Hte* [*argU* *ileY* *leuW* Cam^R^]) strain (Agilent, California, USA) was transformed with these plasmids. Recombinant proteins were induced in cultures with 1 mM of Isopropil-β-D-1-thiogalactopyranoside (IBI Scientific, Iowa, USA) for 4 h. Cultures were subsequently adjusted to an optical density 600 nm of 1.0. Samples were resuspended in 50 μL sodium dodecyl sulfate (SDS) 2× (Bio-Rad, California, USA) and heated for 5 min at 95°C. The proteins were separated by gel electrophoresis (12% SDS-polyacrylamide gel electrophoresis) and visualized with Coomassie stain (Bio-Rad). Recombinant proteins were finally purified using Nickel-Nitrilotriacetic acid columns according to manufacturer’s recommendations (Qiagen, Hilden, Germany) [20].

The commercial MAP antigen PPA-3 was used for PTb detection (Allied Monitor Laboratory, Missouri, US), which is an immunogenic protoplasmic antigen obtained from bacterial lysates from *Mycobacterium avium*. Finally, the native hapten (NH) from *B. melitensis* was selected for brucellosis diagnosis, a 14.5 kDa polysaccharide that can be used to differentiate vaccinated from naturally infected animals [21, 22].

### Sera

A total of 49 positive and 48 negative samples for *Brucella*, 32 positive and 32 negative samples for MAP and 90 positive and 90 negative samples for SRLV were used. These samples were obtained from the goat sera bank from the veterinary school (UNAM) and were previously tested for detecting antibodies for SRLV, *B. melitensis*, and MAP, through an ELISA commercial Kit (caprine arthritis encephalitis [CAE] virus antibody test kit, VMRD^®^, Washington, USA), the agglutination test (and subsequently confirmed by the complement fixation test), and the agar gel immunodiffusion assay, respectively.

### Standardization of the Luminex^®^ system

Before devising the multiplex assay and establishing the optimum antigen concentrations, an indirect ELISA was used with the negative and positive sera for each antigen. The optimum antigen concentrations per well were also established from the ELISA tests: 20 μg/mL for p16, 3 μg/mL for gp38 (recombinant proteins for CAE), 40 μg/mL for PPA-3 (Allied Monitor Laboratory for MAP), and 1 mg/mL for NH (NH for brucellosis), with conjugate dilutions from 1/20,000 to 1/30,000 (data not shown).

The multiplex xMAP^®^ Luminex^®^ platform (Bio-Plex 200, Bio-Rad) uses polystyrene microspheres that can be coated with antigens or antibodies and that contain a mix of internal fluorophores with individual codes [10]. The xMAP Luminex^®^ reader works as a flow cytometer, in which a first laser identifies the sphere, while a second laser detects the antigen-antibody reaction based on the phycoerythrin (PE) excitation wavelength. For the detection of SRLV, MAP and *B. melitensis*, antigens were attached to different microsphere regions at the previously established concentrations. Adequate coupling of antigens to spheres was verified by recovery rates. Maximum binding (B0) values, as determined by incubation of microspheres with known antigen, sample control of each disease and fluorophore concentrations, was used to standardize the assay. The number of attached molecules was estimated by fluorescence and measured in medium intensity of fluorescence (MIF) units.

Protein antigens were covalently attached to the spheres through carboxylated groups by a two-step carbodiimide reaction protocol. Briefly, the spheres were first activated with ethyl dimethyl aminopropyl carbodiimide (Thermo Fisher Scientific, Massachusetts, US) to induce a reaction that causes the carboxylated groups on its surface to form an unstable intermediary (o-acylisourea), which was then stabilized with nihydroxysulfosuccinamide (Thermo Fisher Scientific). This stabilized intermediary is then capable of reacting with the primary amines of the protein of interest [23, 24]. For coupling of the non-protein NH of *B. melitensis*, the sphere was modified with adipic dihydrazine acid (Thermo Fisher Scientific) and the antigen was oxidized with sodium (SP) (J.T. Backer, Massachusetts, US) to provide an amino group to bind to the carboxyl moiety present in the surface of the sphere and allow NH to attach to the spheres covalently [25].

Antigen coupling efficiency was determined by quantifying the number of sphere-bound molecules. Briefly, a fraction of spheres with the antigen attached was incubated with a known concentration of positive control sera, and the biotin-marked conjugate (that reacts with streptavidin-PE) was later added to allow for the number of bound molecules to be estimated by fluorescence [23]. Recovery rates of attached spheres were then calculated as a coefficient of the final number of spheres × 100/initial number of spheres (an adequate recovery percentage was set as being equal to or over 80%). This procedure was followed for each antigen individually and for the combination of the three, to standardize every reaction in the Luminex^®^ 3-plex assay.

### Validation

Cut-off points, sensitivity, specificity, and plausibility parameters were established to validate the multiplex assay using a “Receiver Operating Characteristic” (ROC) analysis for each independent analyte (Prism 5 GraphPad Prism® software version 7.05.237 for Windows, La Jolla California, USA). In total, 49 positive and 48 negative samples were used to test *B. melitensis* with the NH; 32 positive and 32 negative samples were used to evaluate the MAP assay (with the commercial PPA-3 antigen), 90 positive and 90 negative samples with the p16 SRLV antigen, and 61 positive and 61 negative samples with the gp38 SRLV antigen.

For the validation of the multiplex assay, a positive goat serum for each disease (as a pool) and a negative serum for all diseases were used.

Repeatability, calculated with the intra-assay coefficient of variation (CV) in one trial using positive control sample (PCS) and negative control sample (NCS) pool sera and Reproducibility estimated by three different assays ran in triplicates in 2 days, also using positive and negative control pool samples allowed to determine assay precision. A CV equal to or lower than 25% for both repeatability and reproducibility parameters was considered adequate.

## Results

A total of 200–300 spheres per well were determined as the optimum number to use for each antigen when individually assessed. Conjugate dilutions were 1/20,000, for SRLV, 1/30,000 for MAP, and 1/25,000 for *B. melitensis*.

Optimized conditions for the multiplex assay were established by determining maximum binding (B0) percentages of positive control pool sera that were found to be similar as those used for individual antigen tests. A total of 800–1200 spheres per well (200–300 for each antigen) and a conjugate dilution of 1:25000 were used to obtain the maximum binding expressed as MIF (Table-1).

**Table-1:** Percentages of recovery rates of antigen-coupled spheres and maximum binding expressed as MIF values.

Antigen	Recovery %	Maximum binding (MIF)
SRLV p16	98	8047
SRLV gp38	80	13001
MAP PPA-3	98	6411
*B. melitensis* NH	84	4439

MIF: Medium intensity fluorescence, SRLV=Small ruminant lentivirus, *B. melitensis=Brucella melitensis*, NH=Native hapten, MAP: *Mycobacterium avium* subsp. paratuberculosis

### Luminex assay validation

**Figure-3:**
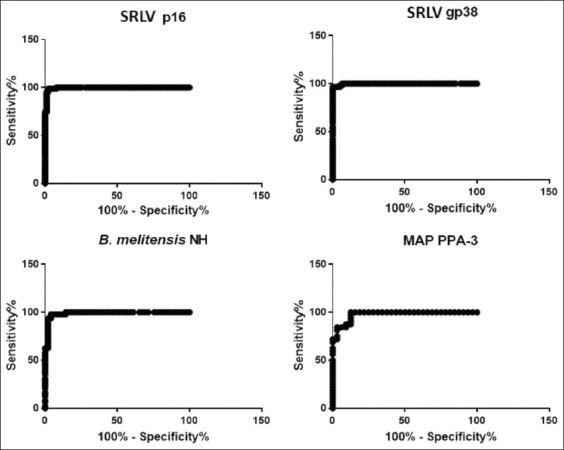
Receiver operating characteristic curve analysis. The cut-off points were established by distribution of positive and negative controls to determine areas under the curve for SRLV (p16 and gp38), *B. melitensis* (NH), and MAP (PPA-3). SRLV=Small ruminant lentivirus, *B. melitensis=Brucella melitensis*, NH=Native hapten, MAP: *Mycobacterium avium* subsp. paratuberculosis.

**Figure-4:**
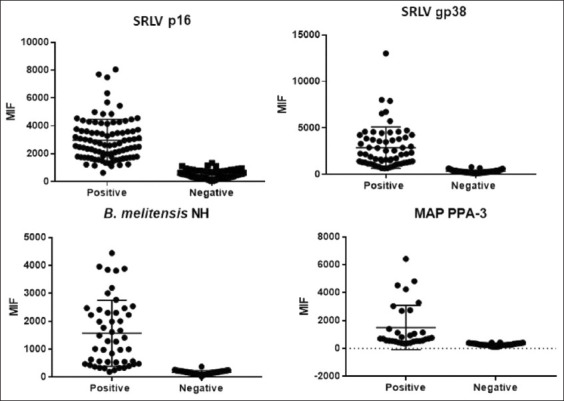
Medium intensity fluorescence analyses. Distribution of positive and negative control samples to establish the cut-off points for antigens (p16, gp38, PPA-3, and NH). SRLV=Small ruminant lentivirus, *B. melitensis=Brucella melitensis*, NH=Native hapten, MAP: *Mycobacterium avium* subsp. paratuberculosis.

A ROC curve analysis [26] was performed for all four antigens. The areas under the curve showed detection of true positive cases (Figure-3). The distribution of PCS and NCS are shown in Figure-4. Cut-off points for the four different antigens were determined with positive and negative control sera and are shown in Table-2. In addition, the plausibility (likelihood ratio) was >10, indicating that the data are highly significant (Table-2).

**Table-2:** Validation parameters of the 3-plex assay for SRLV, MAP, and *B. melitensis* antibody detection.

Antigen	r (%cv)	R (%cv)	CP	S (%)	Sp (%)	LR	n
SRLV p16	(+) 16.1, 20.5, 11.5 (−) 8.2, 7.5, 9.6	(+) 20.2 (−) 21.4	1149	98.89	97.78	44.5	90(+) 90(−)
SRLV gp38	(+) 19.3, 10, 18.1 (−) 14.9, 13.9, 20.6	(+) 16.7 (−) 22.3	659.5	96.72	98.36	59	61(+) 61(−)
MAP PPA-3	(+) 12.5, 20.3, 4.8 (−) 1.2, 23.8, 4.3	(+) 18.4 (−) 13.4	381.3	84.38	96.88	27	32(+) 32(−)
*B. melitensis* NH	(+) 19.2, 3.6, 2.6 (−) 11.1, 10.3, 9.2	(+) 13.1 (−) 9.5	288.9	97.92	95.92	23.99	49(+) 48(−)

SRLV=Small ruminant lentivirus, *B. melitensis=Brucella melitensis*, NH=Native hapten, MAP=*Mycobacterium avium* subsp. paratuberculosis, CP=Cut-off point, LR=Likelihood ratio, n=Number of samples, r=Repeatability, R=Reproducibility, S=Sensitivity, Sp=Specificity. (+) Positive or (−) Negative status of sera

The maximum coefficient of variation of the assay was 23.8% for the NCS and 20.5% for the PCS. Repeatability was found to be under 25% for both PCS and NCS. The CV % for reproducibility was also under 25% (Table-2).

The 3-plex assay for simultaneous antibody detection against SRLV, MAP and *B. melitensis* showed high sensitivity (84.38%–98.89%) and specificity (95.92%–98.36%) (Table-2).

## Discussion

An effective multiplex assay, based on the Luminex^®^ platform, for the identification of antibodies against three relevant diseases in goats (SRLV infections, Brucellosis and PTb) was devised and validated. The Luminex^®^ system is a highly efficient multiplex platform that can screen several analytes simultaneously and is faster than other diagnostic techniques such as ELISA [10, 11, 13–16, 25]. This method has been used to screen antibodies and cytokines in animals and humans [11, 14–16, 25], complying with all parameters of analytic efficiency (reproducibility, repeatability, analytic range, specificity, sensitivity, and detection limit).

The multiplex assay validated in this study allowed us to clearly differentiate positive and negative control sera for each antigen, indicating that there was no cross-reaction. Furthermore, the specificity and the sensitivity of the assay were >95% and 84% for all antigens, respectively. This is consistent with the previous results where differentiation between two distinct populations of analytes within the same Luminex^®^ assay was possible [11, 13, 14, 16]. To improve the diagnostic sensitivity and specificity of the test, it would be convenient to increase the number of positive and negative samples for each disease as referred by the World Organisation for Animal Health, this would derive in a higher percentage of confidence and increase the validity of the assay [27].

The maximum variation coefficient was 23.8% for the negative and 20.5% for the positive control sera, with high sensitivity and specificity values that demonstrated not only the validity of the assay but also indicated that these parameters were not affected by the inclusion of more than one tested analyte in the assay. Thus, this technique can be used for epidemiological research and surveillance and the establishment of control and eradication programs.

Furthermore, the diagnostic panel could be increased by including other antigens such as *Chlamydia abortus*, causal agent of enzootic abortion, another disease known for generating economic losses among goat producers [1]. Using a multiple diagnostic panels that includes more analytes, costs for producers would be reduced, because different diseases could be monitored. This would make the technique more efficient by decreasing the volume, number of samples, reagents, and time for analysis.

## Conclusion

The Luminex^®^ assay for the simultaneous determination of three diseases in goats (PTb, Brucellosis, and SRLV infections) using 4 antigens (PPA-3, NH, p16, and gp38) proved to be a specific, sensitive, and precise test.

## Authors’ Contributions

HDN, ADR, ED, ADP, and LC: Conceptualization. HDN, ADR, MGA, AVR, IJS, NCV, EN and ADP: Methodology. HDN: Validation and analyses. HDN, AVR, IJS and NCV: data curation. HDN, ADR, MGA, ED, and ADP: Resources. HDN, ADR, MGA, NCV, EN, ADP, LMV, and LC: Original draft prepa­ration. HDN, ADR, ED, NCV, EN, ADP and LC: Writing, review, and editing. ADR, LMV, and LC: Supervision. LMV and LC: Project administration. All authors have read, reviewed, and approved the final manuscript.
